# Transcriptional priming and chromatin regulation during stochastic cell fate specification

**DOI:** 10.1098/rstb.2023.0046

**Published:** 2024-04-22

**Authors:** Alison J. Ordway, Rina N. Helt, Robert J. Johnston

**Affiliations:** Department of Biology, Johns Hopkins University, 3400 N. Charles Street, Baltimore, MD 21218, USA

**Keywords:** stochastic, cell fate specification, transcription, chromatin, fly eye, mouse olfactory system

## Abstract

Stochastic cell fate specification, in which a cell chooses between two or more fates with a set probability, diversifies cell subtypes in development. Although this is a vital process across species, a common mechanism for these cell fate decisions remains elusive. This review examines two well-characterized stochastic cell fate decisions to identify commonalities between their developmental programmes. In the fly eye, two subtypes of R7 photoreceptors are specified by the stochastic ON/OFF expression of a transcription factor, *spineless*. In the mouse olfactory system, olfactory sensory neurons (OSNs) randomly select to express one copy of an olfactory receptor (OR) gene out of a pool of 2800 alleles. Despite the differences in these sensory systems, both stochastic fate choices rely on the dynamic interplay between transcriptional priming, chromatin regulation and terminal gene expression. The coupling of transcription and chromatin modifications primes gene loci in undifferentiated neurons, enabling later expression during terminal differentiation. Here, we compare these mechanisms, examine broader implications for gene regulation during development and posit key challenges moving forward.

This article is part of a discussion meeting issue ‘Causes and consequences of stochastic processes in development and disease’.

## 1. Introduction

Stochastic cell fate specification is essential for some aspects of development [1–4]. Whereas deterministic fate decisions result in reproducible outcomes, stochastic fate mechanisms specify between two or more fates in a non-deterministic (random) fashion [5]. These diverse cell subtypes are crucial during development of many species [1–4]. Some of these decisions are probabilistic, generating consistent proportions of cell subtypes within a tissue [5]. Several studies have focused on the contribution of noisy gene expression during stochastic cell fate decisions. In bacteria, noisy transcription drives stochastic mechanisms that are required for survival. These mechanisms diversify cellular functions to enable antibiotic resistance in *E. coli* [6,7] and increase competence for DNA uptake in *Bacillus subtilis* [8]. In yeast, these decisions are primarily driven by variable rates of transcriptional bursting [9–12]. In single cell organisms, stochastic fate decisions have been linked to both intrinsically and extrinsically noisy gene expression [13]. Noisy transcription also plays a role in diversifying neuronal cell fates in vertebrates, including the zebrafish retina [3]. Stochastic fate specification in metazoans is further complicated by the regulation of transcriptional bursting in specific cell types by chromatin remodeling [1,4,9,12,14–17]. Perhaps due to these complexities, conserved mechanisms controlling stochastic fate specification in metazoans have not been clearly identified.

To explore how transcription and chromatin are coordinated in stochastic cell fate specification, we compare the patterning of neuronal subtypes in the *Drosophila* eye and the mouse olfactory system. For each case, we will first introduce how cells are specified as they develop from undifferentiated precursors to terminal neurons. Then, we compare the mechanisms, highlighting similarities in transcriptional priming, chromatin modifications and terminal differentiation that result in stochastic patterning.

## 2. Patterning the random mosaic of photoreceptors in the fly eye

**Figure 1.**
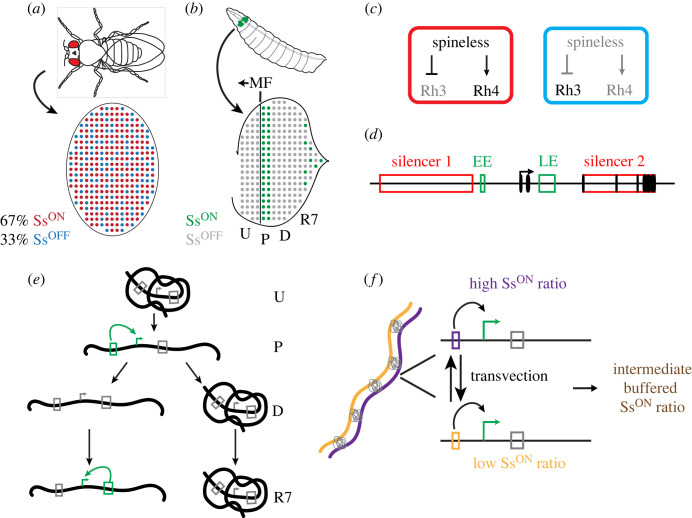
Stochastic R7 subtype fate decision in the fly eye. (*a*) An adult fruit fly and an adult retina displaying the 67% yR7/Ss^ON^ (Rh4, red) to the 33% pR7/Ss^OFF^ (Rh3, blue) ratio. (*b*) A third instar fruit fly larva and the developing eye disc, which will give rise to the adult eye following pupariation. The eye disc is patterned by a wave of morphogenesis called the morphogenetic furrow (MF) that progresses across the developing eye field (arrow indicates direction of movement). Within the developing eye (E), there are four zones of *ss* expression: undifferentiated cells that do not express *ss* (U), R7 precursors expressing *ss* RNA (P), differentiating cells in which *ss* has turned off (D) and terminal R7s in which Ss is expressed in a 67% of R7 cells. Green = Ss^ON^, grey = Ss^OFF.^ (*c*) Ss regulates Rh3 and Rh4. Ss is ON and activates Rh4 in 67% of mature R7s. (*d*) The *ss* locus depicting the exons (black ovals), enhancers (green boxes) and silencers (red boxes). EE, early enhancer; LE, late enhancer. (*e*) Model for *ss* gene regulation. U (undifferentiated), P (precursors), D (differentiating), R7 (terminal R7s). Green boxes indicate active enhancers; grey boxes indicate inactive enhancers. Green arrows indicate promoters that have been activated by the indicated enhancer, while grey arrows indicate inactive promoters. (*f*) Left: Two chromosomes pairing through ‘buttons’ (grey). Right: An example cross between two wild-caught fly strains, one with a high Ss^ON^ ratio and one with a low Ss^ON^ ratio. The progeny of these flies exhibit an average Ss^ON^ ratio.

The *Drosophila melanogaster* eye is composed of approximately 800 ommatidia, or unit eyes. Each of these unit eyes contains eight light-detecting photoreceptor (PR) neurons (R1–R8) [18]. The function of each PR is determined by the expression of light-sensitive rhodopsin proteins (Rhs), responsible for detecting motion or colour [19]. Whereas R1–R6 express Rh1 and detect motion, R7s and R8s express Rh3, Rh4, Rh5 and/or Rh6 and detect colour [19–22]. R7s have two main subtypes: pale R7s (pR7s) and yellow R7s (yR7s) [23,24]. pR7s express UV-sensitive Rh3 and yR7s express a differently tuned UV-sensitive Rh4 [21]. These pR7s and yR7s are randomly patterned in the adult fly eye [25] (figure 1*a*). Though stochastic, this fate choice is probabilistic in that each R7 has a 33% chance of pR7 fate and 67% chance of yR7 fate in wild type flies [23]. Downstream of this fate choice, signaling coordinates R7 and R8 subtypes in the same ommatidium (i.e. pale R7s with pale R8s, yellow R7s with yellow R8s) [26–29]. Pale R8s (pR8s) express blue light-detecting Rh5 and yellow R8s (yR8s) express green light-detecting Rh6 [26–29]. In this review, we focus on the mechanisms that control the stochastic choice between pR7 and yR7 fates.

### (a) Regulation of the pR7 versus yR7 decision

The choice between pR7 and yR7 fates is controlled by the stochastic ON/OFF expression of the PAS-bHLH transcription factor, Spineless (Ss) [30]*.* Ss is expressed in yR7s where it promotes Rh4 expression and yR7 fate, while suppressing Rh3 expression and pR7 fate [30] (figure 1*a,c*). Ss interacts with a network of transcription factors to regulate R7 subtype fate [31–36]. As Ss ON/OFF expression defines R7 subtype fate, we will refer to Rh3-expressing pR7s as Ss^OFF^ R7s and Rh4-expressing yR7s as Ss^ON^ R7s (figure 1*a*).

### (b) Dynamic expression of ss in the developing eye

The adult fly eye and antenna arise from the eye-antennal disc. The eye develops in a wave of morphogenesis, proceeding from the posterior to the anterior (figure 1*b*). Undifferentiated cells in the anterior can be distinguished from differentiating cells in the posterior by a physical indentation called the morphogenetic furrow (figure 1*b*, ‘MF’). During development of the eye, *ss* is initially off in undifferentiated cells (figure 1*b*, ‘U’). As the MF proceeds towards the anterior of the disc, *ss* transcription occurs in the precursor cells posterior to the MF (figure 1*b*, ‘P’). Following expression in precursors, *ss* turns off in differentiating PRs (figure 1*b*, ‘D’) and reactivates later in 67% of terminally differentiating R7s (figure 1*b*, ‘R7’) [14].

### (c) *cis*-regulatory logic controlling ss expression

The dynamic expression of *ss* during development is controlled by two enhancers and two silencers within the *ss* gene locus [2,14,37]. An early-acting enhancer (*early enhancer)* located upstream of the *ss* promoter drives expression of *ss* RNA in precursors, while a late-acting enhancer (*late enhancer*) located within the first *ss* intron drives expression in terminal R7s (figure 1*d*) [2,14,37]. The *early enhancer* and *late enhancer* play distinct roles during R7 subtype specification. The *early enhancer* is sufficient to drive transcription in precursors yet is required for *ss* expression in both precursors and terminal R7s*.* In contrast, the *late enhancer* drives expression only in the terminal R7s. Ablation of the *late enhancer* results in a complete loss of *ss* expression in terminal R7s but does not affect precursor expression [14]. In addition to activation by the *early enhancer* and *late enhancer,* two repressive *silencers* regulate *ss* expression **(**figure 1*d*) [2,14,37,38].

### (d) Chromatin-mediated regulation of ss

During early expression of *ss* in precursors, the Absent, small or homeotic discs 2 (Ash2) and Little imaginal discs (Lid) chromatin modifiers promote *ss* expression. Loss of either of these factors results in a decrease in *ss* expression in precursors and % Ss^ON^ R7s. This role for chromatin modifiers in regulating *ss* expression highlights the importance of the chromatin landscape at the *ss* locus. Initially, in undifferentiated cells, the *ss* locus is inactive and compacted (figure 1*e*, ‘U’). The *early enhancer* then activates expression, and the *ss* locus opens in precursor cells (figure 1*e*, ‘P’). As *ss* RNA but not Ss protein is expressed, transcription appears to ‘prime’ the locus and alter chromatin state. As development continues, *ss* turns off in differentiating cells. It appears that a subset of these differentiating cells retains an open chromatin conformation at the *ss* locus, while the remainder adopts a compacted chromatin state (figure 1*e*, ‘D’). As R7s terminally differentiate, the *late enhancer* reactivates *ss* expression in the subset of cells with open chromatin. In cells with closed chromatin, *ss* remains repressed (figure 1*e*, ‘R7’) [14].

The relationship between transcription driven by the *early* and *late enhancers* and chromatin regulation is complex and dynamic [14]. The *early enhancer* determines chromatin state, as it is required to open chromatin in precursors. In the absence of the *early enhancer,* expression in precursors is lost and the *ss* locus remains compact, preventing activation in terminal R7s by the *late enhancer.* In contrast, the *late enhancer* is susceptible to regulation by chromatin state. Chromatin compaction prevents activation by the *late enhancer* in terminal Ss^OFF^ R7s. The *late enhancer* is functional only in cells with open chromatin. Consistent with this, ablation of the *late enhancer* does not affect chromatin compaction during R7 subtype specification [14]. Taken together, the interplay between chromatin conformation and transcription is necessary for the regulation of *ss* and for the stochastic fate decision in R7 photoreceptors.

### (e) Interchromosomal interactions average the probability of expression

Overlaid on the mechanisms regulating expression at each allele of the *ss* gene, interchromosomal interactions between *ss* loci on homologous chromosomes provide an additional layer of complexity. In most somatic cells in flies*,* homologous chromosomes are held in close proximity (paired) to one another by ‘button loci,’ characterized by TADs and insulator content [39–44]. Pairing enables transvection, in which regulatory DNA elements act between chromosomes to regulate expression of the homologous gene [39,45,46] (figure 1*f*). Transvection enables crosstalk between the regulatory DNA elements at the *ss* locus [37–39]. Communication between alleles occurs during all stages of *ss* expression, including activation by the *early* and *late enhancers* and repression by the *silencers* [38]. Transvection occurs during both the ‘priming’ step early and the chromatin conformation changes at the *ss* locus [38].

The biological roles for transvection have remained largely mysterious. In *Drosophila biarmipes,* transvection regulates a sexually dimorphic pigmentation pattern on the male wing [47]. Studies of *ss* suggested a new biological role for transvection in regulating a probabilistic fate decision [38]. Across 203 wild-caught strains, the majority showed approximately 67% Ss^ON^ R7s similar to the lab strain, suggesting that this is an optimal ratio for colour perception. This proportion is likely selected for, as deviations in % Ss^ON^ R7s alter colour preference [2]. When two wild-derived strains with extreme % Ss^ON^ R7s were crossed, the progeny displayed an intermediate ratio (figure 1*f*). Transvection is not required for stochastic fate decisions, as a fly with only one copy of the *ss* still exhibits the stochastic decision [37,38]. Rather, it appears that transvection buffers the extremes, increasing the population of flies with the optimal approximately 67% Ss^ON^ R7 proportion.

## 3. Stochastic olfactory receptor choice in the mouse olfactory system

**Figure 2.**
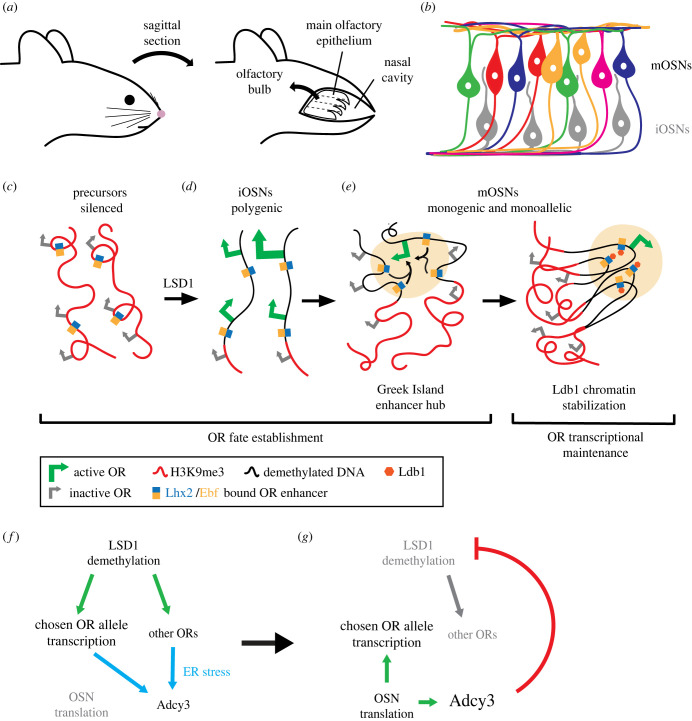
Stochastic olfactory receptor fate decision in the mouse olfactory system. (*a*) In the mouse nasal cavity, the main olfactory epithelium (MOE) consists of olfactory sensory neurons (OSNs) that project their axons to the olfactory bulb of the brain. The OSNs are organized in zones along the dorsal/ventral and medial/lateral axes of the MOE (dashed lines). (*b*) OSNs in the MOE consist of immature OSNs (iOSNs) that do not have a determined OR fate (grey cells), while mature OSNs (mOSNs) stochastically express a single allele of an OR gene within each MOE zone (coloured cells). (*c*) Chromatin and DNA element regulation of OR fate selection using an OR gene cluster on homologous chromosomes as an example. OR expression in precursors is silenced through H3K9me3. Lhx2/Ebf bind to Greek Islands as they resist heterochromatin spreading. Greek Island enhancer hubs are indicated in (*c*–*e*) by blue/orange boxes. (*d*) LSD1 demethylates a small group of OR loci, leading to polygenic transcription. Other ORs remain silenced. One OR allele (large green arrow) may be more highly expressed than the others (small green arrows), according to the ‘winner-takes-all’ model [56,57]. (*e*) Left: The Greek Islands form a multi-enhancer hub, which activates the chosen allele through *cis* and *trans* interactions. Right: Transcription of the chosen allele is maintained through the binding of Ldb1 to Lhx2, which segregates the active promoter into a euchromatic environment while all other inactive alleles remain in heterochromatin. (*f*) Gene regulatory network showing initial OR transcriptional activation prior to OR-mediated feedback. LSD1 demethylation of several OR alleles leads to polygenic transcription. High levels of OR transcription prompts ER stress, pausing OSN translation and upregulating adenylyl cyclase 3 (Adcy3). (*g*) Once Adcy3 accumulates, it represses lysine demethylase LSD1, ceasing the expression of other ORs except for the chosen allele. As the unfolded protein response is relieved, OSN translation is initiated and feeds back to upregulate transcription of the chosen OR and Adcy3.

The mouse olfactory system is comprised of olfactory sensory neurons (OSNs) whose function is determined by the expression of G-protein coupled olfactory receptors (ORs). These ORs are necessary for fine-tuned detection and discrimination of odour molecules and proper axonal targeting to the olfactory bulb of the brain [48–51]. The diversity of ORs in the mouse genome is profound, with 1400 OR genes [48,52,53]. As OSNs in the main olfactory epithelium (MOE) of the nasal cavity differentiate from precursors to immature OSNs (iOSN) and eventually mature OSNs (mOSNs), a single OR allele (maternal or paternal) is expressed, while all other OR genes are silenced [48,52–55]. The choice of receptor is probabilistic across the MOE, with biases in OR selection in zones along the dorsal–ventral and medial–lateral axes (figure 2*a*) [49,50,58–61]. While the precise number of zones is debated, there are an estimated 4–9 overlapping zones of OSNs, with each expressing a group of 50–200 OR genes [49,50,58,59,61]. Therefore, OR expression is organized into zones coupled with stochastic selection of ORs within each zone (figure 2*b*) [49,50,52,58,61]. In this section, we describe the mechanisms controlling OSN fate specification.

### (a) Establishment of OR choice through chromatin reorganization and a multi-enhancer hub

Similar to the R7 subtype fate decision in *Drosophila*, the stochastic choice of mouse OR identity is established through a series of transcriptional priming steps mediated by chromatin reorganization and enhancer interactions between chromosomes. OR genes are organized into genomic clusters, with multiple promoters and intergenic enhancers called the ‘Greek Islands’ spanning the genome [62–64]. Each Greek Island contains binding sites for the LIM domain transcription factors Lhx2 and Ebf, which form a complex and bind to DNA together [65]. In OSN precursors, ORs are silenced by heterochromatin enriched for H3K9me3, creating compact nuclear bodies that conceal most transcription factor binding sites (figure 2*c*) [66–68]. This silencing persists across development in ORs that will not be expressed depending on the MOE zone [1]. However, the Lhx2/Ebf complexes resist this heterochromatin and remain bound to the Greek Islands [63,65].

In iOSNs, chromatin compaction is relieved by the lysine demethylase LSD1, which demethylates H3K9 [67–69]. In regions of open chromatin, Lhx2/Ebf-bound Greek Islands are required to activate polygenic OR transcription. iOSNs lowly express 5–15 ORs across multiple chromosomes [69–72], whereas all other alleles remain inactive (figure 2*d*) [65].

The stochastic selection of the allele for expression in mOSNs is thought to be controlled by a ‘winner-takes-all’ model. In this model, early co-expression of ORs ‘primes’ the decision for singular OR expression later in development [1], with one allele being favored over the others (figure 2*d*, large green arrow) [56]. The priming co-expression of ORs may be sufficient for Greek Island recruitment, whereas one highly transcribed OR allele blocks the expression of other ORs [57]. The favored allele continues its expression, while all other ORs are repressed due to feedback signals or OR mRNA-mediated repression of competing ORs [56,57].

In mOSNs, the allele choice is stabilized through an interchromosomal multi-enhancer hub consisting of Lhx2/Ebf-bound Greek Islands [63–65,73–75]. These Greek Islands form a 3-dimensional hub and activate the allele through intra- and interchromosomal interactions (figure 2*e*) [63–65,73–75]. As a result, OR gene expression in mOSNs is both monogenic and monoallelic [48,52].

### (b) Maintenance of OR choice through DNA elements and protein feedback

Once a single OR allele has been chosen for activation, its expression is maintained for the lifetime of the neuron while the expression of other ORs is repressed. After transcription of a single allele is established, the multi-enhancer hub is stabilized by the LIM domain protein Ldb1, which is recruited by Lhx2 to each Greek Island [76]. Ldb1 interactions on Greek Islands segregate the chosen allele towards euchromatin, while inactive ORs remain silenced in a cluster enriched with H3K9me3 (figure 2*e*) [56,69,71,72,77,78]. These interactions of DNA elements and chromatin ensure continued expression of the selected OR and repression of all other alleles.

In addition to DNA element and chromatin-mediated maintenance, the single OR decision is preserved through OR protein-mediated feedback [55,78–80]. At low frequencies, iOSNs can switch their receptor choice if stable synapses to the brain have not formed or if LSD1 expression is sustained [55]. This switching is terminated once a functional OR protein is expressed, stabilizing its own expression via feedback through the unfolded protein response (UPR) [55,70]. In this feedback mechanism, a group of OR alleles are first transcribed after being demethylated by LSD1, inducing ER stress (figure 2*f*) [69,70]. This activates the UPR, leading to a pause in OSN translation and the upregulation of adenylyl cyclase 3 (Adcy3) (figure 2*f*) [70,81]. Once Adcy3 protein accumulates, it downregulates LSD1, preventing other OR alleles from being demethylated and expressed (figure 2*g*) [69]. As the UPR is relieved, OSN translation is restored to synthesize the functional OR protein, which feeds back to promote transcription of the chosen allele and Adcy3 (figure 2*g*) [69,70]. As a result, OSN receptors stabilize their OR gene fates and do not switch to express other alleles.

## 4. A mechanism for stochastic cell fate specification

By highlighting several shared characteristics between these two examples of stochastic fate specification, we aim to build a framework for a generalizable mechanism consisting of transcriptional priming, changes in chromatin state and terminal gene expression stabilization.

### (a) Transcriptional priming and chromatin conformational changes

In both the fly eye and mouse olfactory system, the stochastically regulated loci are ‘transcriptionally primed’ with a brief pulse of expression in precursor cells prior to their stable expression in terminal neurons. Transcriptional priming is coordinated with changes in chromatin conformation to regulate stochastic fate decisions.

**Figure 3.**
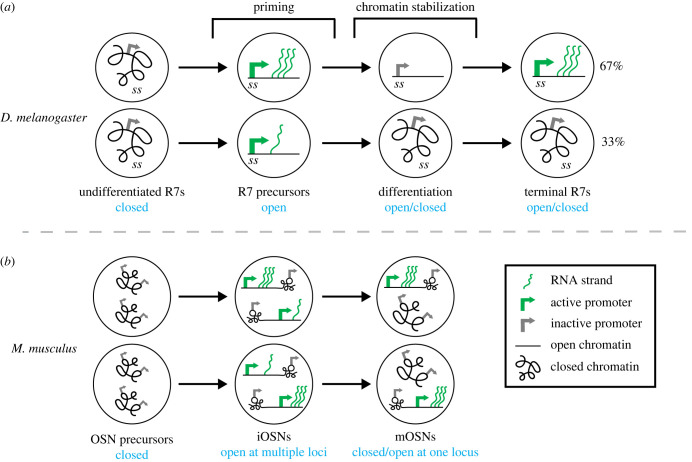
Models of transcriptional priming, chromatin changes and terminal expression. (*a*) In *D. melanogaster*, transcriptional priming opens chromatin in all R7 precursors. During differentiation, *ss* turns off, but some cells retain an open chromatin state (top cell). In terminal R7s, *ss* is re-activated in 67% of R7s with open chromatin (top), while *ss* remains off in the remaining 33% of R7s with compact chromatin (bottom). The stochastic decision may be set in the precursor stage, where some cells could have higher *ss* expression, enabling stable decompaction of chromatin at the *ss* locus (top). (*b*) In *Mus musculus,* transcriptional priming occurs in iOSNs with the expression of multiple ORs with open chromatin. In mOSNs, the expression of a single OR allele persists in each cell, with the active locus having open chromatin and inactive loci having closed chromatin. A highly expressed OR in the iOSN stage may influence the final fate decision.

In the fly eye, the *ss* locus is initially in a compact state in undifferentiated cells. Priming occurs when the *early enhancer* activates *ss* transcription in all R7 precursors, resulting in the recruitment of chromatin regulators and opening of the locus [14,82–84]. The early expression from the *ss* locus ‘primes’ the system and sets up the chromatin landscape for the stochastic decision (figure 3*a*).

In mice, the decision to activate a single OR uses a similar mechanism. Initially, all ORs are compacted in heterochromatin [1,66–68]. In iOSNs, demethylation by LSD1 leads to expression of 5–15 OR genes [67–69]. This early co-expression ‘primes’ the system for the stochastic selection of a single OR later, as one highly expressed OR allele within the group persists and eventually represses competing ORs [56,57,65,69–72] (figure 3*b*).

These two models are similar in that an initial burst of expression ‘primes’ the system, altering the chromatin state and enabling transcription later in development. They differ in how priming is used. In R7 subtype specification in flies, the *ss* locus is primed in all precursor cells and later reactivated in a subset of terminal R7s following changes in chromatin conformation. During mouse OSN specification, multiple OR loci are primed, and later only a single locus remains active in mOSNs following chromatin-level silencing of all other OR loci. The immediate order of priming and chromatin changes also differs, as priming precedes chromatin changes in flies, while chromatin remodeling events occur both before and after priming in mice.

Broadly, transcriptional priming leads to changes in chromatin state that regulate terminal fate specification in both cases. Thus, the interplay between transcriptional priming and changes in chromatin state is an essential step in stochastic cell fate specification in these examples.

### (b) Terminal gene expression stabilization

In both flies and mice, terminal gene expression is maintained and stabilized through chromatin interactions. After ‘transcriptional priming’ in flies, *ss* turns off and the locus compacts in approximately 33% of cells, and remains open in approximately 67% of cells [14]. Cells with open chromatin at the *ss* locus reactivate *ss* and take on the Ss^ON^ R7 fate, while cells with closed chromatin do not express *ss* and take on the Ss^OFF^ R7 fate [14]. This suggests a *closed – open – closed/open* model (figure 3*a*). In mice, a single OR allele stabilizes its expression through Ldb1-mediated chromatin interactions and protein feedback, while all other alleles are silenced and compacted into heterochromatin [1,65,71,72,80,85]. This follows a *closed – open at multiple loci – closed at most loci/open at one locus* model (figure 3*b*).

Key differences lie in the roles of protein-mediated feedback in the final step of fate stabilization. While stochastic *ss* gene regulation is independent of protein feedback in the fly, transcription network feedback ensures proper expression of Rh4 in *ss*-expressing R7s [14,32]. In mice, OR feedback is a vital mechanism for OR fate maintenance and OSN maturation, preventing co-expression or fate switching of multiple ORs [55,70,79,80]. The mRNA of a dominant OR allele may additionally prevent co-expression [57], which does not occur in the fly eye. These dissimilarities are likely due to the differences in the complexities of these cell fate choices. As the PR fate choice in flies depends on the regulation of one transcription factor's gene locus, simple enhancer- and chromatin-mediated regulation is sufficient to maintain stable expression. With the immense complexity of the mouse olfactory system, feedback by the selected OR appears to have evolved to ensure repression of other OR gene loci.

### (c) Robustness of patterning in stochastic cell fate decisions

In addition to the diversification of cellular functions, stochastic fate mechanisms generate probabilistic proportions of cell types to ensure robustness (i.e. consistency across individuals) in tissue patterning. Consistent ratios and spatial organization of cell types appear essential for optimal sensory perception. In flies, the pattern of R7s in the eye varies between flies and even between the eyes of a single fly, yet each fly of the same genotype has the same ratio of R7 subtypes [23]. In mouse OR selection, several zones of the MOE are restricted in their ability to express certain OR genes [49,50,58,59,61], leading to a probabilistic organization of OR subtypes.

Interchromosomal interactions also play important roles in providing robustness. In flies, homologous chromosome pairing brings alleles of *ss* into proximity, enabling activating and repressing transvection. This interchromosomal regulation is not required for stochastic fate specification, as a single copy of *ss* can make the decision in the absence of other copies. Transvection between two alleles yields an average percentage of Ss^ON^ R7s from the proportion generated by each allele alone, buffering extreme ratios and increasing the population of flies with optimal ratios of photoreceptors. In mice, the enhancers near OR clusters on different chromosomes interact with each other to form the Greek Islands complex [63–65,73–75], which drives monogenic and monoallelic expression of an OR gene [48,52]. The monoallelic activation of a single OR allele coupled with negative feedback ensures that alleles with disruptions in their coding sequences will not be expressed [55,79,80]. In contrast to flies, this interchromosomal interaction in mice is required for transcriptional activation of ORs during differentiation [65].

## 5. Discussion

While the fly visual and mouse olfactory systems have several unique features, they both use transcriptional priming, chromatin state regulation and activation in terminal neurons for stochastic fate specification (figure 3). In both examples, transcriptional priming dictates the chromatin state during a precursor stage that ultimately determines the terminal fate of the neuron. As our understanding of the mechanisms controlling stochastic fate specification is still developing, we highlight stochastic cell fate decisions in other contexts that share hallmarks of this mechanism.

### (a) Fluctuations in HIV-1 Tat transcription control a lysogeny/latency decision

During the HIV-1 life cycle, infected lymphocytes lyse or become latent in a stochastic manner based on the expression of the transactivator of transcription (Tat), an HIV-1 protein [86]. If Tat is highly expressed, the cell will lyse, while low expression leads to latency and cell division [86,87]. This decision is likely regulated by the chromatin state of the region into which the HIV genome has integrated [88], with compact chromatin yielding low-level expression and open chromatin promoting high levels of expression and lysis. This lysogeny/latency decision shares basic principles with the mechanisms in the mouse and fly, involving the interplay of transcription and chromatin.

### (b) T-cell specification is controlled by transcriptional priming and chromatin changes

In the mouse immune system, a subset of haematopoietic progenitor cells is restricted to the T-cell lineage and they later terminally differentiate into T-cells based on the expression of the transcription factor Bcl11b [89–92]. During T-cell differentiation, several transcription factors are expressed that promote T-cell fate, yet the activation of Bcl11b expression is delayed by several days [93]. During this delay, T-cell precursors proliferate before Bcl11b expression reaches critical levels that drive cells to terminal T-cell fate [93].

The delay in *Bcl11b* activation is controlled by a two-step mechanism in which a chromatin conformational change stochastically opens one or both *Bcl11b* gene loci [89]. Once accessible, transcription factors bind the open enhancer to drive expression of the *Bcl11b* gene [89]. As each allele of the *Bcl11b* gene is expressed independently, subpopulations of cells express *Bcl11b* in a mono-allelic or bi-allelic manner, yielding different rates of proliferation and differentiation from individual parent cells [89].

One model suggests that major chromatin conformational changes at the *Bcl11b* locus are driven by the expression of the *ThymoD* lncRNA gene, located in an intergenic region adjacent to the *Bcl11b* locus [94]. Early transcription of the *ThymoD* lncRNA drives a major chromatin remodeling event at the *Bcl11b* locus, which in turn allows for the activation of *Bcl11b* at one or two loci, through parallel *cis* and *trans* regulatory events [89,94]. This mechanism shares similarities to stochastic fate specification in the fly eye and mouse olfactory system, as a transcriptional priming step alters chromatin conformation to enable expression. In contrast to the fly eye and mouse olfactory system that rely on transcriptional priming at the stochastically expressed genes themselves, T- cell differentiation relies on priming by a lncRNA that controls chromatin and expression state of the neighboring *Bcl11b* gene.

## 6. Conclusion and future questions

In each case of stochastic cell fate specification, transcriptional priming, chromatin conformation changes and terminal expression provide a recurring theme, yet their interplay varies across each context. For example, transcriptional priming opens chromatin in the fly eye, whereas chromatin decompaction is required before priming in the mouse olfactory system. In both models, priming is required to determine whether loci remain open and express or recompact and remain silent. How could transcriptional priming determine the outcome?

In the fly eye, *ss* is expressed in all precursors, yet only in a subset of terminal R7s. Perhaps in this context, transcriptional noise functions to ‘prime’ the *ss* locus, in that expression of *ss* exceeds a threshold in a subset of cells to keep the locus open during differentiation and specify terminal Ss^ON^ R7 fate (figure 3*a*). This hypothesis could be tested by measuring *ss* transcription levels in precursors and tracking these cells across developmental time to determine if they become terminal Ss^ON^ or Ss^OFF^ R7s. Assessing transcription and chromatin compaction simultaneously in individual precursors over time would characterize how levels of *ss* transcription relate to chromatin state during differentiation (figure 3*a*).

In the mouse olfactory system, one OR allele is expressed 100 times more highly than others in iOSNs [56]. It is unclear what molecular mechanisms promote this high expression at one OR allele, considering that the highly expressed OR and other loci are similarly demethylated by LSD1. It is also unknown how a priming threshold is set within the co-expressing group of ORs. Additional questions surround how LSD1 demethylates ORs in zone-specific regions to create the probabilistic spatial organization in the MOE.

Ultimately, variation in molecular processes determines stochastic cell fate choices. How intrinsic and extrinsic noise drive stochastic cell fate specification is not understood. Noisy transcription is important for stochastic cell fate decisions from bacteria [6–8,95] to mammals [3,95,96]. Its link with chromatin conformation changes has been implicated in stochastic expression *in vitro* [97], but this relationship is still poorly understood during development. Beyond intrinsic noise in transcription and chromatin conformation, extrinsic noise can arise from the cellular environment involving signaling and mechanical perturbation [13,98]. How noise from extrinsic sources affects stochastic fate specification during metazoan development remains an open question.

Transcriptional priming and chromatin regulation stochastically pattern tissues in flies and mice yet produce stereotyped patterning in other developmental contexts such as sensory neuron subtype specification in *C. elegans* [99,100]. In *C. elegans,* priming occurs following a pre-existing cell lineage decision, resulting in two subpopulations in a deterministic pattern [100]. By contrast, in flies and mice, diverse cell types arise from a homogenous pool of precursors [1,14,56,57]. Are these mechanisms distinct? Or do they share an evolutionary origin? Answering these questions will not only improve our understanding of stochastic fate decisions, but also provide new ideas about the evolution of fate choices during development.

The molecular mechanisms controlling the interplay between transcriptional priming and chromatin state that regulate cell fate remain mysterious. We envision two main models. Transcription priming could have *cumulative effects*. If a gene has high total expression over time, the locus maintains the open chromatin state by evicting histones and/or stabilizing a DNA looping state that enables expression in terminal cells. Alternatively, transcriptional priming may control cell fate decisions through a *transient mechanism*. Noisy transcription may have to surpass a particular threshold, as in bacteria [8], to trigger the open chromatin state and expression in terminal cells. In this model, transcriptional priming would only have to transiently surpass this threshold during the precursor stage of development to open chromatin. In a variation of this idea, chromatin state may be dictated by transcriptional priming precisely during the time window when cells exit the precursor stage (i.e. high transcription yields open chromatin, low transcription yields closed chromatin). Studies of the temporal dynamics of these molecular mechanisms will determine how transcriptional priming and local chromatin environments interact to specify cell fates during development.

## Data Availability

This article has no additional data.
